# Fog-Adaptive-YOLO: A lightweight model for insulator defect detection

**DOI:** 10.1371/journal.pone.0351054

**Published:** 2026-06-11

**Authors:** Xiaoyuan Jin, Yuzhen Zhao, Wangyu Shen, Zhun Guo, Jianjing Gao, Baoxi Yuan, Xiyuan Zhu

**Affiliations:** 1 Shaanxi Key Laboratory of Liquid Crystal Polymer Intelligent Display, Technological Institute of Materials & Energy Science (TIMES), Xijing University, Xi’an, China; 2 School of Electronic Information, Xijing University, Xi’an, China; Nanjing Forestry University, CHINA

## Abstract

Insulator defect detection under foggy conditions suffers from complex backgrounds, small targets, weak features, and severe weather interference, remaining a challenging task for UAV-based inspection. To address these issues, this paper proposes Fog-Adaptive-YOLO, a lightweight fog-adaptive detection network. The FogEnhance module suppresses fog noise and enhances weak defect features; the C3MSGR and C2fMSGR modules optimize lightweight multi-scale feature extraction and aggregation. Experimental results show that on the self-constructed InsDef-Fog dataset, the proposed model achieves 65.4% mAP50 with only 2.74M parameters. It obtains 60.3% mAP50 on the public IDID_FOG dataset and 80.2% mAP50 on the real-world WM-FOG dataset. The model also maintains stable precision on the cross-scene RTTS foggy dataset. These results demonstrate that Fog-Adaptive-YOLO achieves a favorable balance between detection accuracy and lightweight efficiency, well-suited for practical foggy insulator defect detection tasks.

## 1. Introduction

As core components of power transmission lines, insulators provide both electrical insulation and mechanical support. Long-term exposure to harsh environments causes performance degradation and structural defects, posing major risks to power system safety and reliability. Critical hazardous defects include glass loss, polymer damage, polymer contamination, double-glass breakage, physical damage, cracks, flashover, and snow accumulation [1]. Each defect directly impairs transmission line reliability, making timely and accurate identification essential for effective maintenance. Unaddressed defects may trigger regional power outages and significant economic losses in severe cases.

With advances in smart grid and image detection technologies [2,3], traditional manual insulator inspection is being replaced by UAV-based approaches, which are cost-effective, flexible, and operationally efficient [4,5]. UAV inspection uses imaging devices to capture aerial insulator images for subsequent analysis. However, mainstream detection methods prioritize defect recognition while neglecting environmental interference—particularly weather-induced distortions in UAV-captured images. This oversight causes severe performance degradation in practical scenarios. To address this, we introduce fog-adaptive preprocessing to enhance environmental robustness and ensure reliable power system operation. This paper presents Fog-Adaptive-YOLO, a lightweight fog-adaptive detection framework that integrates dedicated modules for fog suppression, efficient feature extraction, and multi-scale feature fusion. It achieves superior performance in foggy conditions while satisfying lightweight deployment requirements. The main contributions of this work are as follows:

1. Aiming at the core challenges of strong fog interference, weak defect features, and insufficient multi-scale feature fusion in foggy insulator defect detection, this paper proposes the Fog-Adaptive-YOLO framework, which is well-suited for UAV inspection scenarios.
2. Designing the FogEnhance module, which integrates multi-scale fusion, optimized fog-aware gating, and edge perception to suppress fog noise and enhance weak features, while achieving lightweight design via channel compression and residual connections.
3. Proposing the C3MSGR module with a triple-nested architecture, combining GhostConv, optimized channel control, and adaptive residual connections to efficiently extract deep foggy defect features without parameter redundancy or shallow feature loss.
4. Developing the C2fMSGR module with a three-stage “splitting-enhancement-fusion” pipeline, realizing full integration of shallow and deep features through progressive aggregation, and embedding anti-fog optimization to improve defect feature discriminability.

The structure of this paper is as follows: Chapter 2 provides a brief overview and review of the research status of insulator defect detection. Chapter 3 elaborates on the core design and implementation details of the FogEnhance module, C3MSGR module, and C2fMSGR module. Chapters 4 and 5 introduce the experimental environment configuration and parameter settings, and present and validate the results of multiple experiments conducted on the self-constructed InsDef-Fog dataset, IDID_FOG dataset, RTTS dataset and WM-FOG dataset. Chapter 6 provides a comprehensive summary of the research work in this paper and discusses potential directions for future related research.

## 2. Related work

Early methods for insulator defect detection primarily relied on manual inspection. However, manual inspection is labor-intensive, requiring operators to traverse mountainous regions, navigate rugged terrains, and endure extreme weather conditions such as severe cold and intense heat.

During early technological development, insulator defect detection mainly depended on traditional image processing techniques. Zhao et al. [6] proposed an insulator localization method integrating directional angle and binary shape prior knowledge to address localization challenges. Liao et al. [7] developed an approach fusing local features with spatial order information from insulator images to enable overhead insulator detection.

Yang et al. [8] presented a systematic review on the resilience assessment of urban integrated transportation networks, which summarized relevant theoretical connotations, classified evaluation indicator systems from the perspectives of network topology and element coupling, and comprehensively reviewed mainstream quantitative assessment methods including numerical analysis, simulation modeling and data-driven techniques. Huang et al. [9] proposed a diagnostic framework integrating Geographically Weighted Regression and Random Forest interpretation to investigate the spatiotemporal evolution of the mismatch between urban traffic crash risks and safety governance, using a decade of crash data from a rapidly urbanizing Chinese city.

In recent years, advancements in deep learning theory have significantly improved image recognition accuracy through the application of Convolutional Neural Networks (CNNs). This technology has been widely adopted across diverse fields. Object detection algorithms are currently categorized into two paradigms. Two-stage methods include R-CNN [10], Fast R-CNN [11], Faster R-CNN [12], and Mask R-CNN [13]. Zhai et al. [14] proposed a hybrid knowledge domain-based CNN (HK R-CNN) to address challenges in small target detection and limited training data, achieving a detection mAP of 79.27%. Zhao et al. [15] enhanced Faster R-CNN by integrating the Feature Pyramid Network (FPN) with an adaptive HSV color space thresholding algorithm for image segmentation, yielding mAP values of 90.8% and 91.7% for glass and composite insulators, respectively. One-stage algorithms are exemplified by the YOLO series [16–24]. Liu et al. [25] developed MTI-YOLO, a lightweight insulator detection network with a Spatial Pyramid Pooling (SPP) module, optimized for complex aerial imagery to improve accuracy and feature representation for specific insulator sizes. Song et al. [26] introduced an improved Deformable YOLOX variant, which effectively enhances detection of dense small defects in insulator inspection scenarios. Yang et al. [27] proposed a depth-aware and domain-adaptive network based on the Visual State Space Model (VSSM), integrating appearance, motion and 3D depth information with domain adaptation constraints to mitigate day-night domain shift in traffic crash detection. It achieved a recall of 96.043%, an F1-score of 97.003% and a real-time inference speed of 118 FPS.

To address adverse weather challenges in UAV-based power line inspection, multiple YOLO model enhancements have been proposed. PGE-YOLO [28] employs a cross-scale feature aggregation framework, enabling robust defect detection on transmission lines under variable meteorological conditions, particularly for small defects in UAV imagery. TL-YOLO [29] combines an ODConv backbone with bidirectional FPN and multi-scale attention modules to improve accuracy for debris detection in complex weather. Fusion-Net [30] integrates attention mechanisms and memory units to accelerate inference and boost foreign object detection during severe weather, demonstrating strong performance in UAV inspections. Liu et al. [31] developed YOLO-FOD, a lightweight real-time detection model tailored for object detection under fog, snow, rain and other adverse weather. It designed OSDBB-ELAN, SPD-DSC and GISM-DSC modules together with the F-EIoU loss to strengthen multiscale feature fusion and bounding box regression, which greatly reduces computational complexity and achieves superior mAP performance on challenging weather datasets. Zhang et al. [32] proposed CIA-YOLO, an enhanced multi-type insulator fault detection algorithm based on YOLOv8, to address image degradation under complex weather and insufficient small-target detection accuracy. It introduces the CPA-Enhancer adaptive image-processing module, replaces the C2f Bottleneck with the IMSD-Block multi-scale feature fusion structure in the backbone, and adopts the AR-FPN module in the neck, achieving a mAP of 94.5% on the self-constructed MTIF-CWE multi-weather insulator dataset with minimal parameter increase. Weather-Domain Transfer-Based Attention YOLO [33] introduces a weather domain synthesis architecture that standardizes insulator image processing across weather conditions, reducing domain shifts and improving defect detection consistency under varying environmental scenarios.

Based on the above issues, this paper proposes a lightweight object detection model named Fog-Adaptive-YOLO, aiming to balance detection accuracy and efficiency, thereby meeting the application requirements of actual transmission line detection scenarios.

## 3. Method

### 3.1 FogEnhance

The FogEnhance module is developed to improve the detection accuracy and robustness of insulator defect detectors in foggy environments, while integrating channel simplification to achieve a lightweight design. This module attains superior performance through the integrated optimization of multi-scale fusion, fog perception gating, insulator defect edge detection, and residual connections. It also addresses a critical design limitation in existing fog mask generation methods by effectively utilizing input features.

To tackle the multi-scale characteristics of insulator defects and the challenge of small defects being obscured under foggy conditions, the module adopts a multi-scale pooling fusion strategy. This strategy combines global adaptive average pooling with 3 × 3 and 5 × 5 local average pooling layers. The pooled outputs are processed through spatial mean operations to standardize feature dimensions. Subsequently, the multi-scale pooled features are concatenated along the channel axis to integrate global and local information, with the core concatenation process detailed in equation (1).


$$\begin{array}{c} X_{multi}=Concat(X_{gap},X_{lap\text{3}},X_{lap\text{5}}) \end{array}$$ (1)

Here, X_multi_ denotes the fused feature after multi-scale pooling aggregation. Concat(⋅) represents the channel-wise concatenation operation used to integrate multi-scale feature information. X_gap_ is the output feature of global adaptive average pooling, while X_lap3_ and X_lap5_ correspond to the outputs of 3 × 3 and 5 × 5 local average pooling, respectively. The feature dimension ℝ^B×3C×1 × 1^ indicates the batch size B, channel dimension 3C, and spatial resolution of 1 × 1.

The FogEnhance module incorporates an optimized fog perception and modulation mechanism to suppress fog-induced interference and implement effective fog-aware gating control. This mechanism leverages global pooling features to distinguish foggy regions from insulator regions, thereby dynamically generating a fog mask. This enables the precise separation of fog-induced interference and defect features.

Additionally, an edge perception mechanism is integrated to enhance defect detectability. Edge features of cracks and loose components are extracted via deep convolution, and an edge mask is generated to amplify critical defect characteristics. The module employs a dual strategy combining channel pruning optimization and 1 × 1 convolution-based compression to minimize computational load. Following fog control modulation and multi-scale feature fusion, a lightweight fully connected layer performs feature transformation and preliminary attention weight calculation. Residual connections are then applied to mitigate feature degradation. Global pooling features undergo dimension alignment via 1 × 1 convolutions, which are subsequently fused with preliminary weights through superposition to generate the final channel attention weights. The core process is outlined in equation (2).


$$\begin{array}{c} W_{ca}=Sigmoid\;\left(X_{fc\text{2}}+\overset{bias=False}{\underset{\text{1}\times \text{1}}{Conv}}\left(X_{gap}\right)\right) \end{array}$$ (2)

In this equation, W_ca_ denotes the final channel attention weight. Sigmoid(·) normalizes the attention weight into the range of [0, 1]. X_fc2_ represents the initial attention feature generated by 1 × 1 the fully connected layer, and Conv^bias=False^ denotes the 1 × 1 convolution without bias, which is adopted for feature dimension alignment and residual information compensation.

**Fig 1:**
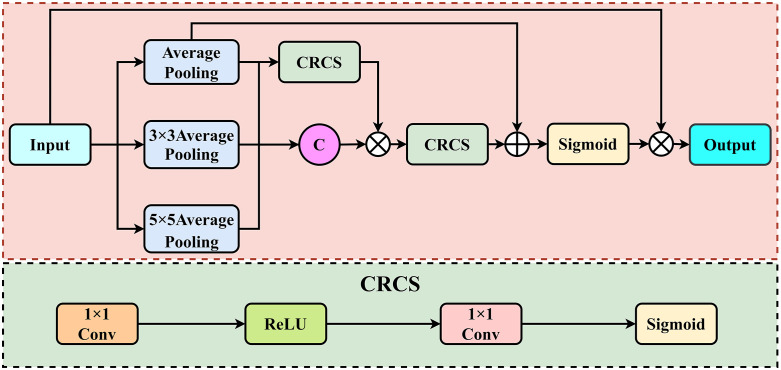
Structure of FogEnhance.

Finally, the input feature map is modulated through attention weighting to enhance defect features and suppress interference from foggy conditions. The final enhanced feature map is output, which can effectively improve the insulator defect detection performance of the subsequent detector in foggy environments. Fig 1 illustrates the detailed structure of the FogEnhance module.

### 3.2 C3MSGR

The C3MSGR module is designed to improve the accuracy of feature extraction and real-time inference efficiency for foggy insulator defect detection, while maintaining an optimized lightweight structure. It adopts a three-layer nested architecture and integrates multiple optimization strategies, including channel coefficient optimization, lightweight convolution selection, and collaborative optimizations with the FogEnhance module. This ensures feature enhancement and fog resistance while reducing model parameters and computational complexity.

To address the difficulties in extracting insulator defect features and the degradation of shallow features under foggy conditions, the C3MSGR module employs a top-level dual-branch integration strategy. It constructs a parallel structure consisting of a feature enhancement branch and an original feature branch, both of which use GhostConv for channel compression. The optimized channel ratio coefficient λ determines the number of channels in each branch, and the outputs of the two branches are concatenated and fused via 1 × 1 convolutions to restore the original channel dimensions.

The C3MSGR module incorporates the MSGABottleneck module to achieve in-depth feature extraction of insulator defects under foggy conditions. This bottleneck module adopts a dual MSGAConv structure, implementing a process of channel compression, feature enhancement, and channel restoration. An adaptive residual reduction strategy ensures effective information transmission throughout the process. Input channels are first compressed to C_mid_ via the first MSGAConv layer and then restored to the target channel count through the second MSGAConv layer. A residual branch is integrated to complement features and enhance the model’s representational capacity. The core computation is formalized in equation (3).


$$\begin{array}{c} x_{MSGABottleneck}={MSGAConv}_{\text{2}}({MSGAConv}_{\text{1}}(x))+S(x) \end{array}$$ (3)

Here, x refers to the input feature map of the bottleneck module, and xMSGABottleneck is the final output feature. MSGAConv1 and MSGAConv2 denote the convolution units for channel compression and channel restoration, respectively. S(·) stands for the adaptive residual branch, which supplements shallow feature information and alleviates the gradient vanishing problem.

The optimized channel ratio coefficient λ is a key hyperparameter designed to balance feature representation capability and lightweight complexity. It controls the channel compression ratio in GhostConv, dual-branch fusion, and MSGABottleneck, where the intermediate hidden channel C_mid_ is calculated as C_mid_ = λ·C. Candidate values of λ∈{0.1, 0.2, 0.3, 0.4, 0.5} are adopted, and the optimal value is determined through ablation experiments to achieve the best trade-off among detection accuracy, parameter scale, and inference speed. The specific value of the channel ratio coefficient λ is detailed in Table 10 of Section 5.4 (Ablation Analysis).

**Fig 2:**
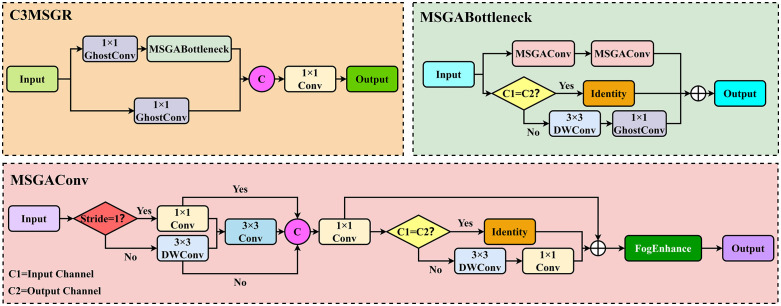
Structure of C3MSGR.

The MSGAConv embedded within the MSGABottleneck module serves as the core computational unit of C3MSGR. It employs multiple optimization strategies and an integrated design with the FogEnhance module, further improving feature extraction efficiency and resistance to fog interference. It first performs channel compression via dynamic lightweight convolution selection, then reduces the number of multi-scale kernels to extract localized defect features. The compressed features are concatenated with locally extracted features and fused with a residual branch before integrating the FogEnhance module for fog enhancement. Fig 2 illustrates the detailed architecture of the C3MSGR module.

### 3.3 C2fMSGR

The C2fMSGR module is developed for foggy insulator defect detection to enhance feature extraction accuracy and real-time inference efficiency under an ultra-lightweight architecture with low parameter redundancy. It adopts a three-stage framework consisting of initial feature splitting, progressive deep enhancement, and multi-stage feature aggregation. The module integrates collaborative optimizations such as channel ratio regulation, lightweight convolution selection, progressive bottleneck enhancement, and embedded anti-fog feature purification. These optimizations retain the abilities of feature enhancement and fog interference suppression while significantly reducing model parameters and computational complexity. Meanwhile, to address the inadequate mining of defect features and loss of shallow information in foggy scenarios, the module determines the core processing channel dimension by optimizing the channel ratio coefficient λ, providing stable dimensional support for subsequent feature splitting and enhancement.

For lightweight initial feature preprocessing, C2fMSGR combines GhostConv lightweight convolution with channel splitting to generate parameter-efficient high-quality feature subsets. The input feature map is first projected into a 2c high-dimensional space via GhostConv to avoid the parameter explosion of traditional convolutions, and then split into two c-channel sub-features along the channel dimension. This design preserves shallow features and provides dual-branch inputs for subsequent progressive enhancement.

For lightweight initial feature preprocessing, C2fMSGR combines GhostConv lightweight convolution with channel splitting to generate parameter-efficient high-quality feature subsets. The input feature map is first projected into a 2c high-dimensional space via GhostConv to avoid the parameter explosion associated with traditional convolutions, and then split into two c-channel sub-features along the channel dimension. This design preserves shallow features and provides dual-branch inputs for subsequent progressive enhancement.

To fully exploit the deep discriminative features of insulator defects under fog interference, the C2fMSGR module incorporates an ordered sequence of n MSGABottleneck modules. Each MSGABottleneck takes the latest feature in the current list as input, and its enhanced output is reinserted into the list to form a forward progressive enhancement chain. This design gradually deepens feature representation and incrementally improves fog interference suppression capability. The progressive enhancement process is formalized in equation (4). The value of n for the MSGABottleneck modules is detailed in Table 11 of Section 5.4 (Ablation Analysis).


$$\begin{array}{c} Y_{extended}=Y\cup {\{m}_{i}(Y[-\text{1}])\;|\;i=\text{1},\text{2},...,n\} \end{array}$$ (4)

In this equation, Y denotes the initial feature list, and Y_extended_ represents the feature set after progressive enhancement. The symbol ∪ denotes the set union operation for feature aggregation, m_i_ (·)means the i-th MSGABottleneck mapping function, and Y[−1] indicates the latest deep feature in the feature list, where n is the stack number of bottleneck modules. This progressive architecture ensures comprehensive extraction of deep features while maintaining implicit associations between deep and shallow features through sequential propagation, effectively preventing loss of weak defect features during enhancement.

To achieve efficient multi-stage feature fusion and target channel restoration, the C2fMSGR module employs a combination of channel concatenation and 1 × 1 convolution. This strategy enables the seamless integration of features from different stages while ensuring dimensional compatibility with downstream detection networks. The feature aggregation and channel restoration process is formalized in equation (5).


$$\begin{array}{c} x=Conv\;\left((\text{2}+n)c,c_{\text{2}},\text{1})(Concat\left(Y_{extended},\text{1}\right)\right) \end{array}$$ (5)

Here, Y_extended_ denotes the enhanced feature set obtained by progressive aggregation. Concat(·) performs channel concatenation on multi-stage features, and Conv((2 + n)c,c_2_,1) represents the 1 × 1 convolution that compresses the channel dimension from (2 + n)c to c. The variable x is the final fused feature output of the C2fMSGR module. This multi-feature aggregation strategy effectively integrates the shallow texture information and deep defect information of insulators, further enhancing the feature discrimination ability in fogged interference environments.

**Fig 3:**
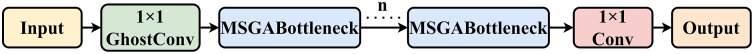
Structure of C2fMSGR.

Through its three-stage efficient architecture, the C2fMSGR module realizes the complete process of lightweight feature preprocessing, progressive deep defect feature extraction, multi-stage feature fusion, and fog interference suppression. The detailed architecture of the C2fMSGR module is illustrated in Fig 3.

### 3.4 Fog-Adaptive-YOLO

**Fig 4:**
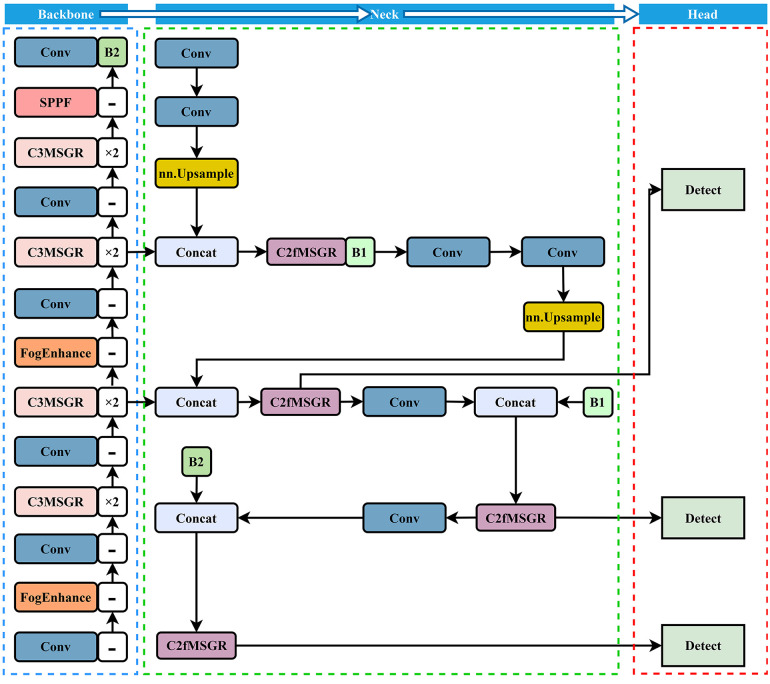
Structure of Fog-Adaptive-YOLO.

To address the core challenges in insulator defect detection under foggy conditions —including strong fog interference, weak defect features, and inadequate multi-scale feature fusion — this study proposes the Fog-Adaptive-YOLO detection network, an improved YOLO-based architecture incorporating three key innovations. First, the backbone network integrates the C3MSGR and FogEnhance modules. The C3MSGR module, featuring a three-level nested architecture and optimized channel compression, achieves lightweight deep feature extraction while reducing computational redundancy and enhancing the expression of insulator defect features. The embedded FogEnhance module suppresses fog-induced noise to highlight discriminative weak defect information. Second, the neck network adopts the C2fMSGR module combined with an “upsampling and cross-layer concatenation” design. The C2fMSGR module progressively aggregates features through a bottleneck enhancement mechanism, integrating the shallow texture details and deep semantic features of insulators. Upsampling and cross-layer concatenation further align multi-scale feature dimensions, improving fusion efficiency. Third, the detection head employs a multi-scale detection mechanism, utilizing scale-specific fused features from the neck network and completing foggy-environment defect detection via an optimized Detect structure to enhance recognition accuracy across defect sizes. The overall architecture of Fog-Adaptive-YOLO is depicted in Fig 4.

All authors declare no potential conflicts of interest. This research does not involve human participants, human data, or animal experiments, thus no ethical approval or informed consent is required.

## 4. Research methods

### 4.1 Dataset description

1) InsDef-Fog: The InsDef-Fog dataset for transmission line insulator defect detection is established based on real-world transmission line infrastructure in a specific region. The dataset construction workflow consists of four core components: physical transmission line insulators, a UAV-based data acquisition platform, predefined cruise routes, and captured insulator image samples.

This work adopts the DJI M300RTK UAV as the flight platform, equipped with a DJI Zenmuse H20T gimbal camera for image collection. The M300RTK provides a 55-minute flight endurance and a 2.7 kg payload capacity, enabling stable aerial photography of insulator defects in field scenarios. Flight routes are rationally planned according to the spatial layout of transmission lines, with a fixed flight altitude of 30 meters above the ground. Route design fully considers line spacing, insulator placement and flight stability to guarantee full image coverage of insulators along power lines. Moreover, the UAV is equipped with automatic obstacle avoidance and high-precision navigation systems, ensuring safe and stable operation in complex field environments.

After manual screening of raw aerial images, a total of 2150 images with various defect types are retained. The dataset covers nine defect categories: glass-dirty, glass-loss, polymer, polymer-dirty, two-glasses, broken, insulator damage, flashover and snow accumulation. The entire dataset is partitioned into training, validation and test sets at a ratio of 7:2:1.

2) IDID_FOG: IDID_FOG is an open-access dataset focusing on foggy transmission line insulator defects, derived from the public IDID_Plus dataset. It contains high-quality annotated insulator images captured under natural foggy conditions, mainly including two typical defect types: flashover and broken damage. The dataset comprises 1456 images, which are divided into training, validation and test subsets following a 7:2:1 ratio.
3) RTTS: The RTTS dataset is a publicly available benchmark for object detection in foggy real traffic scenarios. It consists of high-quality annotated foggy traffic images with five major target categories: pedestrian, bus, bicycle, motorbike and truck. RTTS contains 4322 images and is split into training, validation and test sets at a ratio of 7:2:1.
4) WM-FOG: WM-FOG is a dedicated real foggy insulator defect subset constructed by screening samples from the Weather-Insulator (WI) Insulator Defect Dataset and the Merged Public Insulator Dataset (MPID). It collects high-quality annotated power line insulator images captured under authentic natural foggy weather, covering multiple common insulator defects to support the research of insulator defect detection in real foggy scenarios. The WM-FOG dataset includes 1600 images, divided into training, validation and test sets in a standard 7:2:1 proportion.

### 4.2 Data preprocessing

Transmission line inspection is frequently affected by foggy weather, and UAV-captured insulator images usually suffer from blurring and degraded visual quality, which severely deteriorates defect detection performance. To address this issue, this paper proposes a dedicated fog simulation algorithm based on the atmospheric scattering model [34], which artificially synthesizes foggy samples to enrich data diversity and improve the robustness of the insulator defect dataset.

The proposed fog synthesis algorithm first extracts valid images containing insulator regions according to annotation labels. A customized pixel-level iterative calculation function is then adopted, which integrates image center coordinates, scale parameters and fog density coefficients to simulate natural fog distribution. The mathematical formulation is given as:


$$\begin{array}{c} J(x)\;=\;I(x)\;\times \;t(x)\;+\;A\;\times \;(\text{1}\;-\;t(x)) \end{array}$$ (6)

J(x) denotes the pixel value of the fogged image, I(x) represents the pixel value of the original fog-free insulator image, t(x) indicates the atmospheric transmittance inversely proportional to fog density, and A corresponds to global atmospheric light intensity. The fogging algorithm in this study is optimized based on this model: first, atmospheric light intensity A is fixed at 0.5 to simulate medium-brightness natural fog conditions. Fog effects are centered on the image’s geometric center, with transmittance t(x) calculated using Euclidean distances from each pixel to the center. Three fog concentration coefficients β (0.03, 0.07, 0.12) are defined to simulate mild, moderate, and severe fog levels. Following atmospheric scattering principles, pixel brightness and contrast are adjusted to generate the InsDef-Fog fogged defect dataset. The rationality of the selected A value is further verified through ablation experiments in Section 5.4, with detailed quantitative results presented in Table 14.

Different from conventional uniform fog simulation methods, the proposed algorithm adopts gradient-varying fog concentration to fit real atmospheric visibility changes, effectively enhancing the model’s adaptability to complex fog interference. During data preprocessing, the entire defect dataset is processed in batches via the proposed algorithm, which enriches the meteorological diversity of InsDef-Fog and further strengthens the model’s robustness against fog interference. Finally, the augmented dataset is divided into a 70% training set, a 20% validation set and a 10% test set for subsequent model training and quantitative evaluation.

### 4.3 Evaluation metrics

To comprehensively assess the detection accuracy and real-time operational efficiency of the proposed model, this study employs precision, recall, average precision (AP), mean average precision (mAP), F1 score, and frames per second (FPS) as the core performance evaluation metrics. These indicators evaluate the detector’s performance in object detection tasks from multiple perspectives, covering critical aspects such as detection correctness, target recognition comprehensiveness, and model inference speed. In the evaluation metrics, TP, FP and FN represent true positive, false positive and false negative samples, respectively. P(r) denotes the precision function varying with recall rate r, and the integral from 0 to 1 calculates the area under the precision-recall curve for AP computation. N is the total number of defect categories, and AP_i_ is the average precision of the i-th category. T_m_ denotes the average inference time for a single image, which is used to calculate the FPS value.

1) Precision and Recall: Precision denotes the proportion of truly positive samples among all samples predicted as positive by the detector, which characterizes the accuracy of the model’s positive target judgment. Recall, by contrast, represents the proportion of all actual positive samples successfully identified by the model, primarily measuring the detector’s comprehensiveness in capturing targets. Their specific definitions are given as follows:

$$\begin{array}{c} Precision=\;\frac{TP}{TP+FP} \end{array}$$ (7)

$$\begin{array}{c} Recall=\frac{TP}{TP+FN} \end{array}$$ (8)

2) Average Precision (AP): As a core metric for evaluating object detection model performance, AP reflects the balance between precision and recall when the model detects specific object categories. Its value is obtained by calculating the area under the precision-recall curve for the corresponding category, with the equation defined as:

$$\begin{array}{c} AP=\int_{\text{0}}^{\text{1}}P(r)dr \end{array}$$ (9)

3) Mean Average Precision (mAP): This metric represents the average of AP values across all categories in the dataset, serving to assess the detector’s overall detection performance on the entire dataset. Notably, the “m” here denotes the mAP value calculated when the intersection over union (IoU) threshold between the predicted bounding box and the ground-truth box is set to 0.5. The calculation equation for mAP is:

$$\begin{array}{c} mAP=\frac{\text{1}}{N}\sum_{i=\text{1}}^{n}{AP}_{i} \end{array}$$ (10)

4) F1 Score: The F1 score integrates both precision and recall, defined as their harmonic mean, thereby comprehensively reflecting the model’s overall detection performance. Its calculation equation is:

$$\begin{array}{c} F\text{1}\;score=\text{2}\;\times \frac{Precision\times Recall}{Precision+Recall} \end{array}$$ (11)

5) Frames Per Second (FPS): As a key metric for quantifying model inference speed, FPS refers to the number of frames (or images) processed per second. A higher FPS value indicates stronger real-time detection capability of the model. Its calculation equation is:

$$\begin{array}{c} FPS=\;\frac{\text{1}}{T_{m}} \end{array}$$ (12)

6) Floating Point Operations (GFLOP): As a core metric for evaluating the computational complexity of detection models, GFLOP represents the total number of floating-point operations required for a single image inference. A lower GFLOP value implies fewer computational resource consumption and better lightweight deployment performance.
7) Inference latency: As a vital metric for measuring the real-time performance of detection models, inference latency refers to the time consumed for the model to complete single-image inference. A lower inference latency value indicates faster inference speed and better real-time deployment capability on edge devices such as UAVs.

### 4.4 Training strategies and implementation details

To ensure experimental reproducibility, all experiments were implemented on a unified high-performance deep learning server. The hardware configuration adopts an AMD Ryzen 5 5600 six-core CPU and an NVIDIA GeForce RTX 4060Ti-16GB GPU, with the Windows 11 operating system. The software environment is established on Python 3.10.18, CUDA 11.8, PyTorch 2.3.1, and torchvision 0.18.1.

The image size is uniformly set to 320 × 320 for model training. All key hyperparameters are elaborately configured according to detection task characteristics and hardware limitations. For better readability and experimental repeatability, the main training hyperparameters are summarized in Table 1. The cosine decay strategy is adopted to gradually reduce the learning rate in the late training stage, facilitating stable parameter optimization and detection accuracy enhancement. Meanwhile, reasonable settings of optimizer, momentum, weight decay and training epochs are adopted to accelerate convergence, suppress overfitting, and enhance the generalization ability of the proposed model.

**Table 1:** Training hyperparameter settings.

Hyperparameter	Setting
Input image size	320 × 320
Initial learning rate	1e-2
Learning rate strategy	Cosine decay
Optimizer	Adam
Momentum	0.937
Training epochs	200
Weight decay	5e-4
Batch size	8

## 5. Experiments and analysis

### 5.1 Results on InsDef-Fog and IDID_FOG

Comparison of Fog-Adaptive-YOLO with YOLOv11, YOLOv12, and YOLOv13 series: Compared with the YOLOv11, YOLOv12, and YOLOv13 series, Fog-Adaptive-YOLO exhibits prominent superiority in detection accuracy and computational efficiency for UAV foggy-day target detection tasks, as summarized in Tables 2–4. In terms of detection accuracy, Fog-Adaptive-YOLO achieves an mAP50 of 65.4%, which comprehensively outperforms all lightweight and medium models in the YOLOv11 series, and surpasses YOLOv12n, YOLOv12s and YOLOv12l, while being slightly lower than YOLO12m. It also delivers higher accuracy than YOLOv13s and YOLOv13l, and far exceeds the mAP50 of 51.4% obtained by YOLOv13n. Meanwhile, its parameter count is only 13.67% of YOLOv11m, 10.39% of YOLOv12l, and 9.96% of YOLOv13l. It achieves a competitive FPS of 111.11, which is approximately 2.24 times that of YOLOv11m and 4.37 times that of YOLOv12m. In terms of computational complexity and real-time latency, the GFLOP of Fog-Adaptive-YOLO is only 8.5 G with an inference latency of 6.5 ms, far lower than those of the medium and large models in the three series. Its precision reaches 0.624, outperforming YOLOv11m, YOLOv12m, YOLOv12l, YOLOv13n and YOLOv13s, while maintaining a competitive recall rate and presenting powerful capability of positive sample identification.

**Table 2:** Performance comparison between Fog-Adaptive-YOLO and YOLOv11 series.

Method	Params↓	FPS↑	mAP_50_↑	F1↑	P↑	R↑	GFLOP↓	Inference
								latency(ms)↓
YOLOv11n	2.58	156.25	58.2	0.601	0.613	0.590	6.3	3.4
YOLOv11s	9.41	98.03	61.0	0.642	0.720	0.580	21.3	7.6
YOLOv11m	20.05	49.504	60.4	0.584	0.526	0.658	68.0	18.1
YOLOv11l	25.31	37.31	61.3	0.612	0.627	0.598	87.1	24.2
Fog-Adaptive-YOLO	2.74	111.11	65.4	0.592	0.624	0.564	8.5	6.5

**Table 3:** Performance comparison between Fog-Adaptive-YOLO and YOLOv12 series.

Method	Params↓	FPS↑	mAP50↑	F1↑	P↑	R↑	GFLOP↓	Inference
								latency(ms)↓
YOLOv12n	2.55	120.48	58.7	0.613	0.628	0.600	6.3	5.5
YOLOv12s	9.25	69.93	62.5	0.610	0.634	0.588	21.4	11.8
YOLOv12m	20.13	25.44	69.6	0.606	0.583	0.633	67.6	26.9
YOLOv12l	26.38	23.64	59.5	0.593	0.550	0.644	89.3	39.8
Fog-Adaptive-YOLO	2.74	111.11	65.4	0.592	0.624	0.564	8.5	6.5

**Table 4:** Performance comparison between Fog-Adaptive-YOLO and YOLOv13 series.

Method	Params↓	FPS↑	mAP50↑	F1↑	P↑	R↑	GFLOP↓	Inference
								latency(ms)↓
YOLOv13n	2.45	102.04	51.4	0.510	0.474	0.553	6.2	7.7
YOLOv13s	9.00	144.93	63.6	0.637	0.571	0.719	20.7	5.7
YOLOv13l	27.52	122	63.5	0.635	0.647	0.624	88.1	6.2
Fog-Adaptive-YOLO	2.74	111.11	65.4	0.592	0.624	0.564	8.5	6.5

In comparison with mainstream models of each series, Fog-Adaptive-YOLO achieves an excellent trade-off between detection accuracy and lightweight performance. Its mAP50 not only leads lightweight nano models including YOLOv11n, YOLOv12n and YOLOv13n, but also exceeds the medium and large-scale models YOLOv13s and YOLOv13l. Meanwhile, its parameter quantity and GFLOP of 8.5 G are only a small fraction of those medium and large models, realizing the effective compatibility of high detection accuracy and low computational consumption. Although its inference speed is slightly lower than individual lightweight baseline models such as YOLOv11n, YOLOv13s and YOLOv13l, it possesses obvious advantages in inference latency and computational complexity compared with most medium and large models, and still maintains superior real-time performance than YOLOv13n. The precision of Fog-Adaptive-YOLO exceeds multiple comparison models, and its recall rate is higher than that of YOLOv13n, which effectively reduces missed detection in complex foggy scenarios and improves detection reliability. Nevertheless, its mAP50 is still lower than that of YOLOv12m, indicating that there is still room for further performance optimization in application scenarios with extremely high accuracy requirements.

As shown in Table 3, the YOLOv12 series exhibits an abnormal non-monotonic performance trend as model parameters increase. The mAP50 gradually rises from YOLOv12n to YOLOv12m, reaching a peak of 69.6% at the parameter scale of 20.13 M, while it drops sharply to 59.5% for YOLOv12l with 26.38 M parameters. This anomalous phenomenon is mainly caused by complex fog interference and small-size insulator defect characteristics in the foggy insulator detection dataset. The excessively large network scale of YOLOv12l introduces a large number of redundant parameters, which are prone to overfitting to fog noise and generating feature redundancy, thus seriously degrading detection accuracy.

In contrast, the proposed Fog-Adaptive-YOLO maintains a compact lightweight parameter size of only 2.74 M. It achieves a high mAP50 of 65.4%, which is substantially higher than that of YOLOv12n and YOLOv12l, and also outperforms YOLOv12s in detection accuracy. In terms of computational complexity and real-time performance, the GFLOP of Fog-Adaptive-YOLO is only

8.5 G, which is far lower than YOLOv12s, YOLOv12m and YOLOv12l. Meanwhile, it achieves a high FPS of 111.11 and a low inference latency of 6.5 ms, presenting superior real-time performance compared with YOLOv12s, YOLOv12m and YOLOv12l, with only a slight gap in FPS against YOLOv12n.

To explore the influence of model scale on the detection performance of insulator defects in foggy weather, Fog-Adaptive-YOLO-s, Fog-Adaptive-YOLO-m and Fog-Adaptive-YOLO-l are developed on the basis of the baseline Fog-Adaptive-YOLO by adjusting the network depth factor and width factor. With the gradual increase of depth and width factors, the model parameters and computational complexity rise correspondingly, the feature representation capability is further improved, and the inference latency increases slightly. The constructed multi-scale variants are conducive to analyzing the changing rules of detection accuracy, lightweight performance and real-time inference capability under different model scales. Table 5 intuitively presents the quantitative performance comparison of all the above model variants.

**Table 5:** Performance comparison of different Fog-Adaptive-YOLO variants.

Method	Params↓	FPS↑	mAP_50_↑	F1↑	P↑	R↑	GFLOP↓	Inference
								latency(ms)↓
Fog-Adaptive-YOLO-n	2.74	111.11	65.4	0.592	0.624	0.564	8.5	6.5
Fog-Adaptive-YOLO-s	2.75	105.26	79.5	0.759	0.728	0.793	27.2	7.3
Fog-Adaptive-YOLO-m	5.79	101.01	84.3	0.823	0.805	0.843	55.0	8.5
Fog-Adaptive-YOLO-l	11.65	92.59	89.4	0.853	0.857	0.849	101.7	9.0

**Fig 5:**
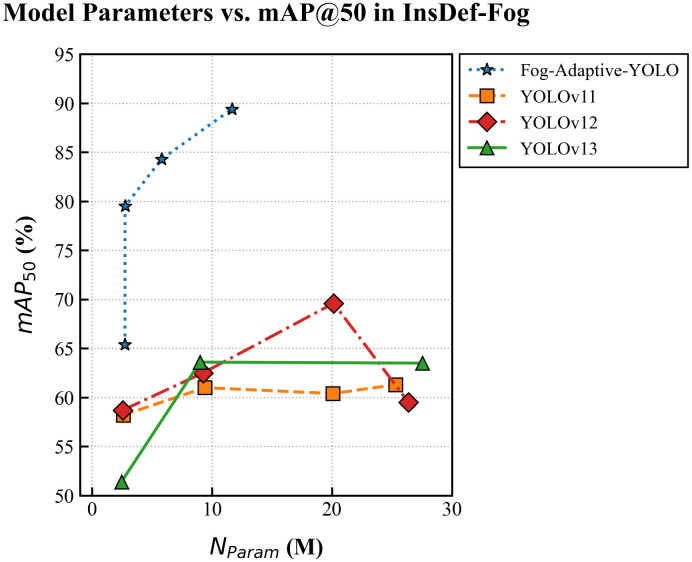
Performance comparison of Fog-Adaptive-YOLO, YOLOv11, YOLOv12 and YOLOv13 series on the InsDef-Fog dataset.

Fig 5 visually presents the mAP50 performance of each method. All Fog-Adaptive-YOLO variants achieve significantly higher mAP50 than YOLOv11, YOLOv12 and YOLOv13 models under similar or even smaller parameters, showing a better accuracy-efficiency trade-off in foggy insulator defect detection. The baseline YOLO series only obtain marginal and unstable performance improvement with increasing model scale, and fail to effectively utilize larger model capacity in foggy degraded scenes due to insufficient environmental adaptability. In contrast, our three Fog-Adaptive-YOLO variants exhibit a steady upward trend, with mAP50 increasing linearly from 65.4% to 89.4% as parameters grow, and even the lightweight Fog-Adaptive-YOLO-n outperforms all compared YOLO models. The rich variant configuration enhances the interpretability of comparative analysis, which fully demonstrates the effectiveness and superiority of the proposed method in foggy insulator defect detection.

Comparison with state-of-the-art methods: To further demonstrate the superiority of Fog-Adaptive-YOLO, it was compared with several state-of-the-art and mainstream lightweight models on the InsDef-Fog and IDID_FOG datasets. The models included in the comparison are: YOLOv5n, YOLOv6n, YOLOv8n, YOLOv10n, YOLOv11n, YOLOv12n, YOLOv13n, Hyper-YOLO [35], Le-YOLO [36], YOLO-TLP [37] and YOLOv8n-SPTS [38]. Tables 5 and 6 record the performance of different methods on InsDef-Fog and IDID_FOG datasets respectively.

**Table 6:** Performance comparison of each model on InsDef-Fog.

Method	Params↓	FPS↑	mAP_50_↑	F1↑	P↑	R↑	GFLOP↓	Inference
								latency(ms)↓
YOLOv5n	2.50	175.43	62.5	0.611	0.599	0.625	7.1	2.8
YOLOv6n	4.23	178.57	49.8	0.512	0.527	0.498	11.8	2.7
YOLOv8n	3.01	172.41	61.6	0.575	0.512	0.657	8.2	3.1
YOLOv10n	2.68	152.7	62.8	0.581	0.612	0.553	6.5	3.3
YOLOv11n	2.58	156.25	58.2	0.601	0.613	0.59	6.3	3.4
YOLOv12n	2.55	120.48	58.7	0.613	0.628	0.6	6.3	5.5
YOLOv13n	2.45	102.04	51.4	0.51	0.474	0.553	6.2	7.7
Hyper-YOLO	3.94	294.12	18.7	0.292	0.307	0.279	10.8	2.2
Le-YOLO	1.89	192.31	26.6	0.391	0.423	0.363	4.36	3.8
YOLO-TLP	1.90	400	53.9	0.563	0.532	0.598	9.2	1.1
YOLOv8n-SPTS	3.15	217.39	17.7	0.303	0.345	0.270	12.4	3.3
Fog-Adaptive-YOLO	2.74	111.11	65.4	0.592	0.624	0.564	8.5	6.5

InsDef-Fog: In terms of mAP50 Fog-Adaptive-YOLO outperforms YOLOv5n, YOLOv8n, YOLOv10n, YOLOv11n, YOLOv12n, YOLOv13n, Hyper-YOLO, Le-YOLO, YOLO-TLP and YOLOv8n-SPTS and achieves the best detection accuracy among all comparison models. Fog-Adaptive-YOLO reaches an FPS of 111.11 which is higher than YOLOv13n Hyper-YOLO and YOLOv8n-SPTS. It obtains a GFLOP of 8.5 and an inference latency of 6.5 ms. Its computational complexity is lower than YOLOv6n Hyper-YOLO and YOLOv8n-SPTS. Its inference latency is higher than most ultra-fast lightweight models but lower than YOLOv13n. This indicates that Fog-Adaptive-YOLO maintains superior detection accuracy and achieves competitive performance in parameter quantity computational complexity and inference speed.

Le-YOLO and YOLO-TLP show better real-time performance in FPS and inference latency. Le-YOLO consumes fewer computational resources but its mAP50 is 38.8% lower than Fog-Adaptive-YOLO. YOLOv8n-SPTS presents the lowest detection accuracy and its parameter quantity and computational complexity are both higher than Fog-Adaptive-YOLO. These lightweight models sacrifice detection accuracy excessively to pursue high inference speed and cannot meet the practical requirements of foggy insulator defect detection. All comparison results are presented in Table 6.

Overall Fog-Adaptive-YOLO achieves an excellent tradeoff between accuracy computational complexity and inference speed. The designed FogEnhance C3MSGR and C2fMSGR modules effectively improve feature extraction and detection ability for foggy insulator images. Although the model cannot reach the extreme real-time performance of individual lightweight methods it owns obvious advantages in detection accuracy parameter scale and computational consumption.

IDID_FOG: In terms of mAP50, the Fog-Adaptive-YOLO achieves competitive performance, closely matching the top-performing models in the comparison. Its mAP50 of 60.3% is slightly lower than YOLOv8n’s 61.8% but surpasses YOLOv6n, YOLOv10n, YOLOv11n, YOLOv12n, YOLOv13n, Hyper-YOLO, and Le-YOLO. With a parameter count of 2.74M, it is more compact than most compared models, including YOLOv8n, YOLOv10n, and YOLOv8n-SPTS. Its FPS of 227.27 outperforms Hyper-YOLO, Le-YOLO, and YOLOv13n, demonstrating excellent real-time performance. In terms of computational complexity, its GFLOP of 8.5 is lower than YOLOv6n, Hyper-YOLO, and YOLOv8n-SPTS, reducing resource consumption. Its inference latency of 2.7ms is better than YOLOv13n, Hyper-YOLO, and Le-YOLO, ensuring efficient real-time response.

The YOLOv10n and YOLOv6n achieve higher frame rates but at the cost of lower detection accuracy, with mAP50 values of 55.9% and 50.8% respectively. Models with fewer parameters such as Le-YOLO and YOLOv13n have lower mAP50 values, reflecting the trade-off between lightweight design and detection performance. Table 7 presents the detailed performance metrics of each model, including parameter count, FPS, mAP50, F1 score, precision, recall, computational complexity, and inference latency.

**Table 7:** Performance comparison of each model on IDID_FOG.

Method	Params↓	FPS↑	mAP_50_↑	F1↑	P↑	R↑	GFLOP↓	Inference
								latency(ms)↓
YOLOv6n	4.23	400	50.8	0.507	0.505	0.508	11.8	0.7
YOLOv8n	3.01	357.14	61.8	0.653	0.703	0.61	8.1	0.8
YOLOv10n	2.68	714.29	55.9	0.618	0.651	0.589	6.5	1.0
YOLOv11n	2.58	322.58	58.4	0.618	0.66	0.581	6.3	1.0
YOLOv12n	2.55	294.12	56.9	0.607	0.649	0.57	6.3	1.5
YOLOv13n	2.45	181.82	54.9	0.581	0.617	0.548	6.2	3.8
Hyper-YOLO	3.94	151.52	54.4	0.551	0.557	0.545	10.8	3.9
Le-YOLO	1.89	112.36	54.4	0.571	0.578	0.564	4.36	5.0
YOLOv8n-SPTS	3.15	135.13	61.2	0.605	0.635	0.598	12.4	4.5
Fog-Adaptive-YOLO	2.74	227.27	60.3	0.622	0.656	0.592	8.5	2.7

Overall, the Fog-Adaptive-YOLO achieves a well-rounded balance between detection accuracy, computational efficiency, and real-time performance. While it does not reach the absolute highest speed or accuracy among all compared models, it delivers consistent and reliable performance across key metrics, making it a practical and effective solution for foggy environment insulator defect detection tasks.

### 5.2 Analysis of AP performance by categories

To further analyze the performance differences of the model in detecting different types of targets, this section conducts a statistical and comparative analysis of the category AP values of the InsDef-Fog and IDID_FOG datasets. The experimental results are shown in Table 8. The proposed Fog-Adaptive-YOLO exhibits significant differences in its performance across different types of targets. Overall, it shows better detection results for regular and high-quality targets, while the performance for severely degraded and small targets is relatively weaker.

**Table 8:** Analysis of AP performance in InsDef-Fog and IDID_FOG categories.

Categories	InsDef-Fog	IDID_FOG
	mAP_50_	mAP_50_
glass-dirty	40.6	\
glass-loss	46.1	\
polymer	72.1	\
polymer-dirty	44.8	\
two-glasses	99.2	\
broken	54.7	98.8
insulator damage	92.5	\
flashover	62	21.9
snow	77	\

In the InsDef-Fog dataset, target detection performance varies significantly across different categories. The two-glasses category achieves the highest AP value of 99.2%, benefiting from its distinct morphological characteristics and minimal interference from fog, which facilitates effective feature extraction. The polymer category yields an AP of 72.1%, while the snow category records an AP of 77%, demonstrating stable detection for typical targets with clear features. The glass-loss category achieves an AP of 46.1%, and the glass-dirty and polymer-dirty categories reach AP values of 40.6% and 44.8%, respectively.

Such performance differences are mainly caused by the inherent category imbalance in real-world inspection, where rare defects are difficult to collect and show weak features under fog. These results indicate that detection performance declines with increasing fog interference and target degradation, reflecting the model’s sensitivity to complex weather conditions. The broken category achieves an AP of 54.7%, and the insulator damage category reaches an AP of 92.5%, which shows that the model performs well in detecting intact insulator structures but is limited by insufficient samples for rare and partially damaged defects.In the future, we will supplement rare defect samples and adopt class-balanced strategies to further improve detection performance.

For targets in the IDID_FOG dataset, the performance gap between categories is significant. The broken category achieves the highest AP value of 98.8%, benefiting from clear target features and sufficient samples. The flashover category yields an AP of 21.9%, showing relatively weak performance. This discrepancy arises because the flashover targets are small and easily affected by background confusion, leading to reduced recognition accuracy in complex fog scenarios.

Lower AP values occur mainly for targets with small sizes, serious occlusion, or strong fog interference, resulting in weak feature discriminability. This reflects the model’s limitations in detecting highly degraded and tiny targets under heavy fog.

### 5.3 Generalization experiment

To evaluate the adaptive capability and cross-scene generalization robustness of Fog-Adaptive-YOLO in real complex foggy environments, and make up for the limitations of self-built InsDef-Fog and IDID_FOG insulator datasets (single fog type and simple simulated fog generation), the public foggy RTTS dataset was selected for generalization verification. It should be noted that the RTTS dataset focuses on pedestrian and vehicle detection, which is significantly different from transmission line insulators in geometry and background; this experiment mainly assesses the model’s anti-interference ability and generalization performance under real foggy scenes, rather than comparing the same-type target detection accuracy. Detailed performance comparison results of each model on the RTTS dataset are shown in Table 9.

**Table 9:** Performance comparison of each model on RTTS.

Method	Params↓	FPS↑	mAP_50_↑	F1↑	P↑	R↑	GFLOP↓	Inference
								latency(ms)↓
YOLOv5n	2.50	333.33	60.2	0.613	0.713	0.533	7.1	0.8
YOLOv6n	4.23	256.41	57.3	0.598	0.716	0.51	11.8	0.8
YOLOv8n	3.01	294.11	61.2	0.581	0.732	0.53	8.2	0.9
YOLOv10n	2.68	769.23	59.4	0.604	0.713	0.522	6.5	0.9
YOLOv11n	2.58	303.03	58.5	0.599	0.705	0.522	6.3	1.0
YOLOv12n	2.56	312.5	57.7	0.603	0.705	0.523	6.3	1.3
YOLOv13n	2.45	232.55	55.7	0.579	0.701	0.49	6.2	2.6
Hyper-YOLO	3.94	217	63.1	0.624	0.692	0.568	10.8	2.9
Le-YOLO	1.88	185.18	62.5	0.619	0.701	0.543	4.36	3.8
YOLOv8n-SPTS	3.15	200	63.3	0.607	0.659	0.581	12.4	3.4
Fog-Adaptive-YOLO	2.74	211	58.6	0.6	0.713	0.517	8.5	3.3

In terms of mAP50 accuracy, the Fog-Adaptive-YOLO reaches 58.6%, which is slightly higher than that of YOLOv11n and YOLOv12n, and significantly outperforms other comparative models. Its GFLOP is 8.5, which is lower than that of YOLOv6n, Hyper-YOLO and YOLOv8n-SPTS, effectively reducing the consumption of computing resources. The inference latency of the Fog-Adaptive-YOLO is 3.3 ms, which is better than that of Le-YOLO and YOLOv8n-SPTS, ensuring efficient real-time response.

In terms of lightweight property and inference efficiency, the Fog-Adaptive-YOLO has 2.74M parameters, which is more compact than YOLOv6n and YOLOv8n-SPTS. Its FPS reaches 211, which is higher than that of Hyper-YOLO, Le-YOLO and YOLOv8n-SPTS. Combined with its appropriate GFLOP and inference latency, it achieves a good balance between detection accuracy and real-time performance, fully demonstrating the superiority of the fog-adaptive design in practical application scenarios and effectively verifying the strong generalization ability of the model in real fog environments.

To evaluate the cross-scene generalization robustness of Fog-Adaptive-YOLO in real complex foggy environments, and make up for the limitations of self-built InsDef-Fog and IDID_FOG insulator datasets, the real foggy WM-FOG insulator dataset was selected for generalization verification. Different from the controlled simulated fog in self-built datasets, WM-FOG contains natural non-uniform fog, complex illumination variations and real scene noise, which is more consistent with the actual foggy power line inspection scenarios. This experiment aims to fully assess the model’s anti-interference ability and generalization performance under authentic foggy conditions. Detailed performance comparison results of each model on the WM-FOG dataset are shown in Table 10.

**Table 10:** Performance comparison of each model on WM-FOG.

Method	Params↓	FPS↑	mAP_50_↑	F1↑	P↑	R↑	GFLOP↓	Inference
								latency(ms)↓
YOLOv5n	2.50	333.33	73.1	0.698	0.762	0.644	7.1	0.8
YOLOv6n	4.23	238.09	75.2	0.726	0.812	0.656	11.8	0.9
YOLOv8n	3.01	384.61	80.6	0.762	0.846	0.693	8.2	0.9
YOLOv10n	2.68	666.66	76.6	0.734	0.829	0.659	6.5	1.1
YOLOv11n	2.58	294.11	76.4	0.729	0.866	0.63	6.3	1.2
YOLOv12n	2.56	208.33	76.2	0.728	0.788	0.677	6.3	3.0
YOLOv13n	2.45	263.15	68.5	0.666	0.769	0.588	6.2	2.4
Hyper-YOLO	3.94	204.08	45.1	0.550	0.538	0.563	10.8	3.2
Le-YOLO	1.88	117.64	41.0	0.498	0.496	0.5	4.36	5.9
YOLOv8n-SPTS	3.15	102.04	39.9	0.485	0.615	0.4	12.4	6.4
YOLO-TLP	1.90	263.15	72.6	0.683	0.73	0.642	9.2	1.3
Fog-Adaptive-YOLO	2.74	204.08	80.2	0.737	0.852	0.649	8.5	2.1

In terms of mAP50 accuracy, Fog-Adaptive-YOLO reaches 80.2%, which is only slightly lower than YOLOv8n’s 80.6% and outperforms all other competing models. It also achieves competitive results in precision, recall and F1 score. Many lightweight models with high FPS suffer obvious accuracy drop on the real foggy WM-FOG dataset. By contrast, the proposed model maintains superior detection performance in real cross-scene foggy environments, which verifies that the designed modules can effectively suppress real fog noise and enhance weak defect features.

In terms of lightweight performance, computational complexity and inference latency, Fog-Adaptive-YOLO has a moderate parameter scale of 2.74M and a competitive FPS of 204.08. Its GFLOP is 8.5, lower than YOLOv6n, Hyper-YOLO and YOLOv8n-SPTS, which reduces computational resource consumption. The inference latency is 2.1 ms, outperforming YOLOv12n, YOLOv13n, Hyper-YOLO, Le-YOLO and YOLOv8n-SPTS. Although several ultra-light models achieve faster inference speed, they sacrifice detection accuracy severely. Overall, Fog-Adaptive-YOLO achieves a favorable trade-off among detection accuracy, lightweight scale, computational complexity and real-time inference capability, showing strong generalization adaptability to natural complex foggy scenarios.

### 5.4 Ablation analysis

To evaluate the effectiveness and rationality of each improved module, comprehensive ablation experiments were conducted on the InsDef-Fog validation set based on the baseline model with different combination strategies. The quantitative experimental results are presented in detail in Table 11. Each improved component contributes to the detection accuracy, model complexity or inference speed to varying degrees.

**Table 11:** Performance comparison of baseline combinations under different strategies.

Method	FogEnhance	C3MSGR	C2fMSGR	Params	mAP_50_	FPS	P	R
	–	–	–	2.58	58.2	156.25	0.613	0.59
	√	–	–	2.59	61.6	192.3	0.73	0.561
Baseline	–	√	–	2.03	59.5	192.3	0.602	0.573
	–	–	√	1.93	60.3	185.18	0.63	0.555
	√	√	√	2.74	65.4	111.11	0.624	0.564

Specifically, after introducing FogEnhance alone, the mAP50 of the baseline model is increased by 3.4%, with only a slight growth in parameters, while the FPS is significantly promoted to 192.3 and the precision is greatly improved to 0.73. With the addition of C3MSGR only, the parameter count is reduced to 2.03M, and the mAP50 and recall are increased to 59.5% and 0.573 respectively, with the FPS raised to 192.3. Adopting C2fMSGR individually further decreases the parameters to 1.93M, and enhances the mAP50 and precision to 60.3% and 0.63 respectively, while maintaining a high inference speed of 185.18 FPS.

Ultimately, compared with the baseline method, the full model combined with FogEnhance, C3MSGR and C2fMSGR obtains the optimal overall performance, where the mAP50 is remarkably improved by 5.4%, and the precision and recall remain at competitive levels. However, integrating multiple improvement modules together introduced additional computational overhead,resulting in a decrease in FPS.

Table 12 reports the ablation results of different channel ratio coefficient λ. With the gradual increase of λ, the channel expansion degree of each lightweight module rises continuously, leading to a monotonic increase in model parameters and GFLOPs. Meanwhile, the inference FPS decreases steadily due to the growth of computational burden. In terms of detection accuracy, mAP50 rises rapidly at first and reaches the peak value of 65.4% at λ = 0.3, and then gradually declines as λ continues to increase.

**Table 12:** Ablation study of channel ratio coefficient λ.

Channel ratio λ	Params	GFLOP	mAP_50_	FPS
0.1	2.21	8.2	54.8	130.21
0.2	2.47	8.3	59.6	122.45
0.3	2.74	8.5	65.4	111.11
0.4	3.68	8.8	62.3	102.04
0.5	3.85	9.0	58.7	95.24

It can be observed that an overly small λ brings excessive channel compression, which weakens the feature representation ability of the model and results in low detection accuracy. When λ exceeds 0.3, continuously expanding the channel width introduces plenty of redundant parameters and computational overhead, which cannot further improve the detection performance but degrade the real-time inference speed. Consequently, λ = 0.3 is selected as the optimal channel ratio coefficient, which achieves a promising trade-off among detection accuracy, lightweight property and inference efficiency.

Table 13 presents the ablation results of different stacking numbers n of MSGABottleneck. As the stacking number increases from 1 to 4, the model parameters and GFLOPs keep rising continuously, while the inference FPS decreases steadily from 111.11 at n = 1 to 98.04 at n = 4. In terms of detection accuracy, the mAP50 reaches the highest value of 65.4% at n = 1, and gradually declines with the increase of network depth. In addition, from the perspective of training efficiency, the training time for models with n = 2, 3, and 4 is approximately twice as long as that for n = 1, further increasing the overall training cost.

**Table 13:** Ablation study of stacking number n for MSGABottleneck.

Bottleneck number n	Params	GFLOP	mAP_50_	FPS
1	2.74	8.5	65.4	111.11
2	3.83	8.9	58.3	105.26
3	3.91	9.0	55.5	102.04
4	4.00	9.2	55.2	98.04

In the foggy insulator detection task, thick fog interference and tiny defect targets bring complex background noise. Increasing the stacking number of MSGABottleneck cannot bring performance gain; instead, it causes feature redundancy and overfitting to fog noise. Meanwhile, more stacked layers introduce extra computational overhead, reduce real-time performance, and prolong training time significantly—the training efficiency of n = 2, 3, and 4 is roughly half that of n = 1. Therefore, n = 1 is determined as the optimal stacking number to balance detection accuracy, lightweight design, inference speed and training efficiency.

To verify whether the fixed atmospheric light intensity A = 0.5 introduces data bias and affects model performance, we conduct ablation experiments with different values of A, including 0.3, 0.5, and 0.7. These values cover the typical range of natural fog brightness from relatively dim to relatively bright conditions, allowing us to evaluate the impact of A on detection performance under controlled settings. The results are summarized in Table 14.

**Table 14:** Ablation study of atmospheric light intensity A.

Atmospheric light A	Params	GFLOP	mAP_50_
0.3	2.74	8.5	53.1
0.5	2.74	8.5	65.4
0.7	2.74	8.5	53.8

To verify the rationality of our adopted fixed atmospheric light intensity A = 0.5 and eliminate potential data bias, we conduct ablation experiments with three typical values corresponding to different fog brightness conditions: A = 0.3 for dim fog, A = 0.5 for medium fog, and A = 0.7 for bright fog. All experiments adopt the same model structure and test settings, so the model parameters and computational complexity remain unchanged to ensure experimental fairness.The results in Table 14 show that the model achieves the highest mAP50 of 65.4% when using A = 0.5, significantly outperforming the 53.1% obtained with A = 0.3 and the 53.8% obtained with A = 0.7. This is because medium-brightness fog provides the optimal balance between visual realism and defect feature distinguishability. Dim fog at A = 0.3 weakens defect visibility, while over-bright fog at A = 0.7 reduces feature contrast, both leading to performance degradation. These results confirm that A = 0.5 is the optimal setting for fog synthesis in this work.

### 5.5 Visual analysis

**Fig 6:**
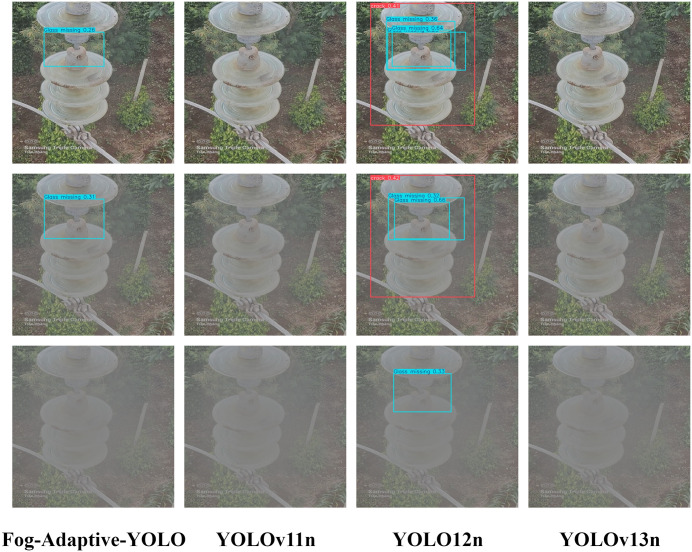
Some detection results of different models on the InsDef-Fog dataset. (The visualization images in this figure are generated based on the original images from the InsDef-Fog dataset used in this study. The InsDef-Fog dataset is self-constructed, with access link: https://data.mendeley.com/drafts/t5f92tv3sh. These images include algorithm-generated detection bounding boxes, confidence score labels, and manually annotated red and blue boxes that highlight representative detection comparison regions. The original InsDef-Fog dataset images are raw, unannotated aerial photographs, so the presented visualization images are similar in scene content but not identical to the original dataset images, and are therefore for illustrative purposes only.).

Fig 6 compares the insulator defect detection results of Fog-Adaptive-YOLO, YOLOv11n, YOLOv12n, and YOLOv13n in mild, moderate, and severe foggy environments. In the mild fog environment, Fog-Adaptive-YOLO can accurately locate the defect targets and fully annotate the bounding boxes, with excellent detection performance; while YOLOv11n and YOLOv13n do not detect any defects, and YOLOv12n has the problem of redundant bounding box overlap. In the moderate fog environment, Fog-Adaptive-YOLO can still accurately identify the defects and fully annotate them, maintaining good detection accuracy and completeness; YOLOv11n and YOLOv13n still have no detection results, and the redundant box problem of YOLOv12n still exists, with a decrease in positioning accuracy. In the severe fog environment, except for YOLOv12n, all models are unable to identify the defect targets, and the detection capability is basically ineffective. Overall, Fog-Adaptive-YOLO has significantly better detection performance than YOLOv11n, YOLOv12n, and YOLOv13n in mild and moderate foggy environments, achieving precise identification and annotation of defects; however, in severe fog conditions, its detection performance is limited, and its performance is inferior to YOLOv12n. This limitation will be further optimized in the model’s feature extraction and anti-interference capabilities in the future.

**Fig 7:**
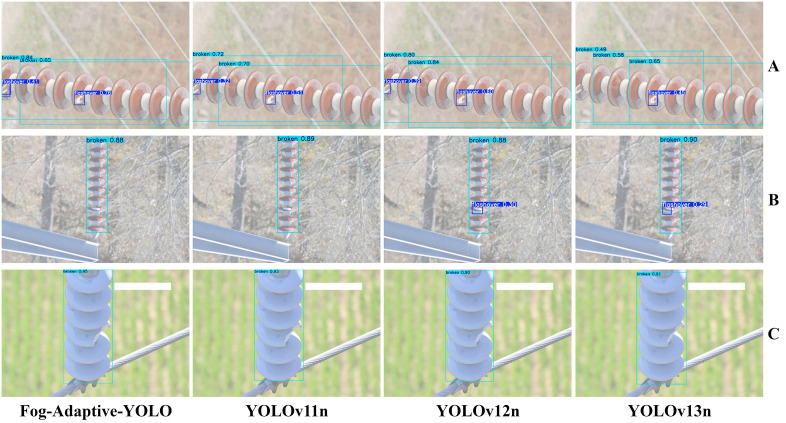
Some detection results of different models on the IDID_FOG dataset. (The visualization images in this figure are generated based on the original images from the public IDID_FOG dataset used in this study. These images include algorithm-generated detection bounding boxes, confidence score labels, and manually annotated blue and green boxes that highlight representative detection comparison regions. The original IDID_FOG dataset images are raw, unannotated aerial photographs, so the presented visualization images are similar in scene content but not identical to the original dataset images, and are therefore for illustrative purposes only.).

Fig 7 presents the visualization results of insulator defect detection for Fog-Adaptive-YOLO, YOLOv11n, YOLOv12n, and YOLOv13n on the IDID_FOG dataset. In scenarios with clear and simple background interference (A, C), Fog-Adaptive-YOLO can accurately locate the defect targets, with complete bounding boxes and the highest confidence level. The detection accuracy and positioning effect of Fog-Adaptive-YOLO are significantly superior to those of YOLOv11n, YOLOv12n, and YOLOv13n, demonstrating excellent defect recognition ability and positioning precision. However, in complex background interference scenarios (B), Fog-Adaptive-YOLO fails to detect the defect targets, while YOLOv12n and YOLOv13n can complete the target recognition. The defect detection performance of Fog-Adaptive-YOLO in most scenarios is better than that of the comparison models, and the overall detection accuracy is the best; however, there is a detection failure problem in specific complex background scenarios, which may be due to the insufficient proportion of complex background scenarios in the training set, and the model’s generalization ability for such scenarios is weak, resulting in detection failure during inference. In the future, we will expand the defect samples in complex background and strong interference scenarios to balance the scene distribution of the training data and improve the model’s scene adaptability.

**Fig 8:**
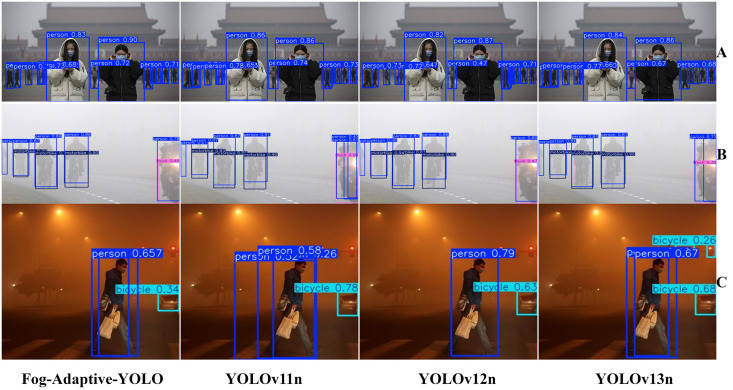
Some detection results of different models on the RTTS dataset. (The visualization images in this figure are generated based on the original images from the public RTTS dataset used in this study. These images include algorithm-generated detection bounding boxes, confidence score labels, and manually annotated blue and green boxes that highlight representative detection comparison regions. The original RTTS dataset images are raw, unannotated aerial photographs, so the presented visualization images are similar in scene content but not identical to the original dataset images, and are therefore for illustrative purposes only.).

Fig 8 shows the visualization results of Fog-Adaptive-YOLO, YOLOv11n, YOLOv12n, and YOLOv13n on the RTTS dataset. RTTS is adopted to make up for the single fog type and simple simulated fog of self-built insulator datasets, focusing on verifying the model’s fog anti-interference and generalization in real unfamiliar scenes rather than same-target accuracy. In scene (A) with fog and dense small pedestrians, Fog-Adaptive-YOLO accurately locates all instances with complete bounding boxes and the highest confidence, significantly outperforming comparison models. In scene (B) of complex foggy roads with multi-scale moving targets, it effectively detects most pedestrians and vehicles with consistent bounding boxes. In night fog scene (C), under low light and dense fog, it stably detects both categories with clear boxes and reliable confidence, while comparison models show lower confidence or incomplete localization, especially for fog- and illumination-affected vehicles.

The visual results confirm Fog-Adaptive-YOLO’s strong cross-scene generalization in real fog environments. Its fog-adaptive structure enables it to adapt to complex natural fog beyond simple simulated fog in insulator datasets. Minor small-target missed detections in extreme scenarios are due to the scene difference between RTTS and insulator inspection, which will be optimized by supplementing more real foggy insulator samples.

## 6. Conclusion

This paper proposes Fog-Adaptive-YOLO, a lightweight fog-adaptive detection network for UAV-based insulator defect inspection under foggy conditions. The network integrates the FogEnhance, C3MSGR, and C2fMSGR modules to suppress fog interference, enhance weak defect features, and realize efficient multi-scale feature learning with low computational cost. Extensive experiments validate the effectiveness and generalization of the proposed method. On the self-constructed InsDef-Fog dataset, Fog-Adaptive-YOLO reaches 65.4% mAP50 with only 2.74M parameters. It achieves 60.3% mAP50 on the public IDID_FOG dataset and 80.2% mAP50 on the real-world WM-FOG dataset, while maintaining robust cross-scene generalization on the RTTS dataset. The proposed model balances detection accuracy, computational complexity, and inference speed, providing a practical solution for foggy insulator defect detection. Despite its promising performance, the model still exhibits limitations in detecting extremely small defects under heavy fog and complex backgrounds. In future work, we will integrate end-to-end defogging preprocessing, adopt multi-modal data fusion, and enhance hard sample mining to further improve the model’s robustness and adaptability in extreme foggy scenarios.
